# Knowledge, attitudes, and practices of university students regarding COVID-19: a cross-sectional study in Vietnam

**DOI:** 10.1186/s12889-022-14442-9

**Published:** 2022-11-03

**Authors:** Dung Anh Doan, Huong Hien Ho, Long Duc Tran, Phuong Lan Nguyen, Anh Thi Lan Le, Dai Xuan Dinh

**Affiliations:** 1 Faculty of Pharmacy, Phenikaa University, Hanoi, Vietnam; 2 Drug Administration of Vietnam, Vietnam Ministry of Health, Hanoi, Vietnam; 3 Department of Pharmacy, National Hospital of Obstetrics and Gynecology, Hanoi, Vietnam; 4 Faculty of Pharmaceutical Management and Economics, Hanoi University of Pharmacy, Hanoi, Vietnam

**Keywords:** COVID-19, Knowledge, Attitude, Practice, University student, Vietnam

## Abstract

**Objective:**

This cross-sectional study investigated the knowledge, attitudes, and practices (KAP) of Vietnamese university students regarding COVID-19.

**Methods:**

A validated questionnaire (Cronbach's alpha = 0.71) was used to survey 1,025 students. A convenience sampling method was used for recruiting students from April to May 2022. The Wilcoxon rank-sum test and the Kruskal–Wallis rank-sum test/Dunn test for multiple comparisons were employed to compare students' KAP scores between two groups and among three groups or more, respectively. Factors associated with students' COVID-19 KAP scores were determined via univariate and multivariate linear regression models. Variables in the multivariate linear regression models were chosen using the Bayesian Model Averaging method in R software version 4.2.0.

**Results:**

A majority of students had good knowledge (75.61%), positive attitudes (98.24%), and good practices toward COVID-19 (94.93%). Regarding the COVID-19 knowledge, the proportions of students who knew that mosquito bites and exposure to/eating wild animals would not lead to COVID-19 infection were not high (47.22 and 34.34%, respectively). More importantly, 70.34% of students thought that vitamins and minerals could help prevent or cure COVID-19. Antibiotics were the first choice for COVID-19 treatment of 438 students (42.73%). Nearly half of students (48.0%) bought antibiotics to keep at home in case of COVID-19 infection. The average KAP scores of medical students (19.97 ± 3.99, 45.10 ± 3.94, 9.72 ± 1.78) and females (18.67 ± 4.44, 44.79 ± 3.79, 9.36 ± 1.84) were significantly higher than those of non-medical students (16.48 ± 4.37, 43.33 ± 4.03, 8.68 ± 1.87) and males (17.01 ± 4.55, 42.79 ± 4.39, 8.77 ± 1.97), respectively (*p* < 0.001). Older students were more likely to have good knowledge and practices than the younger ones (*p* < 0.001). In addition, students using websites of the World Health Organization/the Ministry of Health and scientific articles to seek COVID-19 information were significantly associated with higher KAP scores when compared with those not using these sources (*p* < 0.001, *p* < 0.001, and *p* = 0.00139, respectively).

**Conclusion:**

Students' KAP scores significantly varied by age, sex, major, and sources of COVID-19 information. Although many students had sufficient knowledge, positive attitudes, and good preventive practices toward COVID-19, additional education and training strategies are paramount, especially for non-medical students and males.

**Supplementary Information:**

The online version contains supplementary material available at 10.1186/s12889-022-14442-9.

## Background

Coronavirus disease 2019 (COVID-19) is an infectious disease caused by the SARS-Cov-2 virus. Springing from China in 2019, this virus spread globally and quickly led to the global COVID-19 pandemic [1]. As per the World Health Organization (WHO), as of 11 September 2022, there were 605.71 million COVID-19 confirmed cases and 6.49 million deaths globally. More than 3.13 million new cases and 10,935 deaths were reported from 5 to 11 September 2022 [2]. In Vietnam, as of 21 September 2022, more than 11.46 million cumulative COVID-19 patients, 10.58 million recoveries, and 43,142 deaths were confirmed by the Vietnam Ministry of Health (VMOH) [3]. From December 2021 to April 2022, an outbreak of the COVID-19 pandemic occurred in Vietnam, with tens of thousands of new cases per day [4].

Recently, thanks to the invention and global distribution of many types of COVID-19 vaccines, this pandemic has gradually come under control. Approximately 67.9% of the global population received at least one dose of a COVID-19 vaccine [5]. However, according to WHO, as of 22 May 2022, nearly one billion people in lower-income countries remain unvaccinated. Only 57 countries reached 70% vaccination coverage [6]. In Vietnam, as of 12 September 2022, more than 257 million doses of COVID-19 vaccines were distributed nationwide. 91.95% of the population received at least one dose of a COVID-19 vaccine, and 85.84% were fully vaccinated [7].

In Vietnam, numerous programs and measures were imposed in the entire country to mitigate the outbreak of the COVID-19 pandemic (for example, rigorous in-door quarantine, isolating individuals infected with COVID-19, constraining crowded activities, closing schools and universities, and recommending people to use face masks and hand sanitizers) [8, 9]. To broaden the public's COVID-19 knowledge, the government and the VMOH launched many health education programs, such as sending information on COVID-19 via mobile phone messages, leaflets, and texts to citizens. After a long time of studying via online learning platforms, students have gradually returned to their schools/universities since April 2022. There was a need to assess students' knowledge, attitudes, and practices (KAP) regarding COVID-19 and its prevention, thereby guaranteeing their safety in offline learning. There are many studies conducted to evaluate the KAP of university/undergraduate students regarding COVID-19 all over the world [10–39], including several studies in Vietnam [10, 11]. Most of them were only carried out for medical students. This study investigated the KAP of medical and non-medical university students regarding COVID-19 in Vietnam.

## Methods

### Study design

A cross-sectional, online survey was conducted at Phenikaa University, located in Hanoi capital, Vietnam, during a big wave of the COVID-19 outbreak. In 2022, 5,952 students are learning and studying at this multidisciplinary university.

### The questionnaire

After reviewing and gathering information on COVID-19 from the WHO websites [40] and numerous scientific articles [10–39, 41], a draft of the KAP questionnaire was developed. Two lecturers (from the Faculty of Pharmacy, Phenikaa University) aided the research team in reviewing this questionnaire and checking the plainness and clarity of each question. Then, a pilot study was conducted with the participation of 30 students to check the logic and suitability of the questionnaire (the pre-testing of the questionnaire).

The first part of the final questionnaire included a brief introduction about this study (objectives, procedures), the declaration of anonymity and confidentiality from researchers, and the confirmation of voluntary participation from students. The second part involved 11 questions about the students' personal information (such as sex, age, and major). The last part was the KAP questions/statements (including 16 knowledge statements, 12 attitude statements, and 12 practice statements) (Additional file 1). The overall Cronbach's alpha calculated for a data set of 1,025 students was 0.71, indicating an acceptable level of internal consistency.

### Data collection

The Raosoft sample size calculator (http://www.raosoft.com/samplesize.html) was used to compute the sample size. For the population size of 5,952 students, to achieve a margin of error of 4%, a confidence level of 99%, and a response distribution of 50%, the minimum sample size was 884 students. To increase the validity and generalizability of this research, the researchers endeavored to approach as many students as possible. Because of the COVID-19 pandemic outbreak in Hanoi, participants were recruited using a non-probability convenience sampling technique. The questionnaire designed on Google Forms was sent to students via Facebook, Messenger, or links with Quick Response (QR) codes. From April to May 2022, 1,025 students voluntarily participated in this survey. An estimation of 2,000 students was approached (response rate: about 51.25%). Each student possessed an Edu email given by Phenikaa University (…@st.phenikaa-uni.edu.vn), and they could only answer this questionnaire via these emails.

### Data analysis

After being extracted into a Microsoft Excel file, the data were analyzed using R software version 4.2.0. For the knowledge part, two scores were given for answers of "totally right" to correct statements and "totally wrong" to incorrect statements. One score was given for answers of "probably right" to correct statements and "probably wrong" to incorrect statements. "Do not know" and incorrect answers ("totally right" and "probably right" for incorrect statements/"totally wrong" and "probably wrong" for correct statements) were assigned 0 scores. For the attitude part, scores ranged from 1 (strongly negative) to 5 (strongly positive). For the practice part, good and poor practices were given scores of 1 and 0, respectively. The total score of one student varied from 0 to 32 (knowledge), from 12 to 60 (attitudes), and from 0 to 12 (practices). Higher scores indicated better COVID-19 knowledge, attitudes, and practices. In KAP studies, the cut-off points used to categorize the scores varied. In this study, the KAP scores were dichotomized into "good" and "poor" (for knowledge and practice scores) or "positive" and "negative" (for attitude scores) based on a 50% cut-off point, in line with several KAP studies [42, 43]. The scores ≥ 16 and < 16 indicated good and poor knowledge; ≥ 36 and < 36 indicated positive and negative attitudes; ≥ 6 and < 6 indicated good and poor practices, respectively.

The number (percentage) and mean (standardized deviation) were used to report categorical and numeric variables, respectively. Data normality was evaluated using the *Shapiro–Wilk test* (*p*-value > 0.05 indicating normally distributed continuous variables). The *Wilcoxon rank-sum test* and the *Kruskal–Wallis rank-sum test*/the *Dunn test* for multiple comparisons were employed to compare the KAP scores between two groups and among three groups or more, respectively. Factors associated with students' KAP scores were identified via univariate and multivariate linear regression models. Dependent variables in the multivariate regression models were selected by using the Bayesian Model Averaging method. Statistically significant differences were considered when the *p*-value < 0.05.

## Results

### The general characteristics of students

The participants were predominantly females (69.56%), freshmen/sophomores (79.22%), and students aged from 18 to 20 years old (78.05%). About 30% of students were from Hanoi, and three-fifths lived in rural areas. 60.10% of participants were infected with COVID-19. Only 18.73% of students participated in voluntary activities involving COVID-19. Among students, nearly four-fifths (78.15%) have received at least three doses of COVID-19 vaccines. Social networks and online newspapers were the common sources that most students employed to seek information about COVID-19 (87.61%) (Table 1).

**Table 1:** The general characteristics of the study sample (*n* = 1,025 students)

No	Characteristics		Number	(%)
1	Sex	Male	312	30.44
		Female	713	69.56
2	Age	18	245	23.90
		19	366	35.71
		20	189	18.44
		21	70	6.83
		22 and above	155	15.12
3	Year of study	First	570	55.61
		Second	242	23.61
		Third	83	8.10
		Fourth	71	6.93
		Fifth	59	5.76
4	Major	Medical	494	48.20
		Non-medical	531	51.80
5	Province (home location)	Hanoi	305	29.76
		Namdinh	90	8.78
		Thanhhoa	65	6.34
		Haiduong	59	5.76
		Thaibinh	55	5.37
		Bacninh	53	5.17
		Hungyen	38	3.71
		Bacgiang	37	3.61
		Haiphong	34	3.32
		Others	289	28.20
6	Place of residence	Urban	408	39.80
		Rural	617	60.20
7	The people living with the student	Alone	69	6.73
		Friends	509	49.66
		Family (parents, siblings…)	447	43.61
8	Infected with COVID-19	Yes	616	60.10
		No/don’t know	409	39.90
9	Participated in volunteer activities involving COVID-19 in health facilities	Already	192	18.73
		Never	833	81.27
10	Number of doses of COVID-19 vaccines given	No or 1	10	0.98
		2	214	20.88
		3	784	76.49
		> 3	17	1.66
11	Sources for seeking COVID-19 information	Social networks and online newspapers	898	87.61
		Shares from friends, family members	559	54.54
		Mass media (national news, television…)	675	65.85
		Websites of the World Health Organization, the Ministry of Health, scientific articles	584	56.98

### The COVID-19 knowledge of students

Almost all students (> 95%) were aware of the main ways of COVID-19 transmission (droplets: 98.63%, touching contaminated surfaces: 96.59%) and symptoms (main symptoms of COVID-19 infection: 98.15%, infected with COVID-19 without any unusual symptoms: 88.20%). However, the proportions of students knowing that mosquito bites and exposure to/eating wild animals would not lead to COVID-19 infection were not high (47.22 and 34.34%, respectively). Regarding COVID-19 treatment and prevention, only 10.93% of students knew that hydroxychloroquine could not help prevent infection or death from COVID-19. Antibiotics were the first choice to treat COVID-19 of more than two-fifths of students. Some wrong measures could not help prevent or cure COVID-19, but numerous students thought they could, including using vitamins and minerals (70.34%); adding pepper, ginger, and garlic to food (26.93%); and drinking alcohol, exposure to sunlight or temperatures higher than 25 °C (20.78%) (Table 2). The average knowledge scores of all students were 18.16 ± 4.54 (range: 0–32).

**Table 2:** Detailed responses of university students about COVID-19 knowledge [n (%)]

No	Knowledge questions	Totally right	Probably right	Don’t know	Probably wrong	Totally wrong
1	COVID-19 is a disease caused by the SARS-Cov-2 virus	**773** **(75.41)**	**181** **(17.66)**	37 (3.61)	14 (1.37)	20 (1.95)
2	The virus can be transmitted through droplets produced when an infected person coughs/sneezes/talks	**925** **(90.24)**	**86** **(8.39)**	11 (1.07)	0 (0.00)	3 (0.29)
3	You can be infected with COVID-19 when touching contaminated surfaces, not washing your hands, and then touching your eyes/nose/mouth	**840** **(81.95)**	**150** **(14.63)**	24 (2.34)	7 (0.68)	4 (0.39)
4	The COVID-19 virus can be spread through mosquito bites	149 (14.54)	170 (16.59)	222 (21.66)	**147** **(14.34)**	**337** **(32.88)**
5	Exposure to or eating wild animals can lead to COVID-19 infection	147 (14.34)	233 (22.73)	293 (28.59)	**152** **(14.83)**	**200** **(19.51)**
6	Polymerase Chain Reaction (PCR) can be used to accurately diagnose whether or not a person is infected with COVID-19	**771** **(75.22)**	**207** **(20.20)**	31 (3.02)	11 (1.07)	5 (0.49)
7	Symptoms of COVID-19 infection can include fatigue, cough, fever, shortness of breath, and loss of taste/smell	**876** **(85.46)**	**130** **(12.68)**	17 (1.66)	0 (0.00)	2 (0.20)
8	Unlike the common cold, runny nose and sneezing are less common in COVID-19 patients	**303** **(29.56)**	**358** **(34.93)**	134 (13.07)	145 (14.15)	85 (8.29)
9	A person can be infected with COVID-19 without any unusual symptoms	**591** **(57.66)**	**313** **(30.54)**	48 (4.68)	41 (4.00)	32 (3.12)
10	Patients with chronic medical conditions, the elderly, and the obese have a lower risk of mortality and milder symptoms of COVID-19 infection	169 (16.49)	101 (9.85)	92 (8.98)	**141** **(13.76)**	**522** **(50.93)**
11	Washing hands with soap, using face masks, and limiting crowded gatherings are some effective measures to help prevent COVID-19	**884** **(86.24)**	**116** **(11.32)**	17 (1.66)	6 (0.59)	2 (0.20)
12	Hydroxychloroquine may help prevent infection/death from COVID-19	145 (14.15)	188 (18.34)	580 (56.59)	**62** **(6.05)**	**50** **(4.88)**
13	Antibiotics are the first choice to treat COVID-19	189 (18.44)	249 (24.29)	206 (20.10)	**163** **(15.90)**	**218** **(21.27)**
14	Adding pepper, ginger, and garlic to food does not help prevent and treat COVID-19	**191** **(18.63)**	**271** **(26.44)**	287 (28.00)	176 (17.17)	100 (9.76)
15	Drinking alcohol and exposure to sunlight/temperatures higher than 25 °C can help prevent COVID-19	92 (8.98)	121 (11.80)	226 (22.05)	**201** **(19.61)**	**385** **(37.56)**
16	Vitamins and minerals can help prevent COVID-19 in healthy people or cure COVID-19 in infected people	315 (30.73)	406 (39.61)	165 (16.10)	**71** **(6.93)**	**68** **(6.63)**

### The attitudes of students toward COVID-19

Most students thought that COVID-19 was an extremely dangerous disease (87.80%). Many students would feel very anxious if they, their friends, or their family members were infected with COVID-19 (81.95%) and when watching/reading the news about COVID-19 (69.56%). A high proportion of students would always comply with the epidemic prevention guidelines of the VMOH (96.49%) (for example, wearing face masks: 94.54%, making medical declarations: 95.12%, and quarantining/self-isolating: 94.93%). Furthermore, 878 students (85.66%) were willing to participate in volunteer activities to support the fight against the COVID-19 pandemic. Many students believed the COVID-19 pandemic would be successfully controlled soon (89.37%) (Table 3). The students' average attitude scores were 44.18 ± 4.09 (range: 12–60).

**Table 3:** The attitude of university students about COVID-19 [n (%)]

No	Attitude items	Totally disagree	Disagree	Neutral	Agree	Totally agree
1	COVID-19 is an extremely dangerous disease	5 (0.49)	27 (2.63)	93 (9.07)	287 (28.00)	613 (59.80)
2	I feel very anxious when watching or reading the news about COVID-19	15 (1.46)	59 (5.76)	238 (23.22)	380 (37.07)	333 (32.49)
3	I feel apprehensive and insecure if I, my friends, or my family members are infected with COVID-19	17 (1.66)	48 (4.68)	120 (11.71)	382 (37.27)	458 (44.68)
4	Children and adolescents do not need to take any measures to prevent COVID-19	580 (56.59)	215 (20.98)	32 (3.12)	51 (4.98)	147 (14.34)
5	I will always comply with the COVID-19 prevention guidelines of the Ministry of Health	3 (0.29)	3 (0.29)	30 (2.93)	246 (24.00)	743 (72.49)
6	Wearing face masks is very effective in preventing COVID-19	2 (0.20)	9 (0.88)	45 (4.39)	332 (32.39)	637 (62.15)
7	If infected with the virus, I am ready to go to quarantine at medical facilities or self-isolate at home in case my symptoms are mild	4 (0.39)	9 (0.88)	39 (3.80)	256 (24.98)	717 (69.95)
8	When a person is infected with COVID-19, making a medical declaration is really necessary	5 (0.49)	5 (0.49)	40 (3.90)	270 (26.34)	705 (68.78)
9	I am willing to volunteer at medical facilities to support the fight against the COVID-19 pandemic if necessary	6 (0.59)	8 (0.78)	133 (12.98)	372 (36.29)	506 (49.37)
10	The government should not let overseas Vietnamese return home during the outbreak of the COVID-19 pandemic	67 (6.54)	182 (17.76)	440 (42.93)	155 (15.12)	181 (17.66)
11	Updating COVID-19 information regularly to know how to prevent this disease and avoid misinformation is really necessary and important	3 (0.29)	3 (0.29)	32 (3.12)	257 (25.07)	730 (71.22)
12	I think the COVID-19 pandemic will be successfully controlled soon	4 (0.39)	9 (0.88)	96 (9.37)	335 (32.68)	581 (56.68)

### Students’ practices toward COVID-19

In general, students had good practices toward COVID-19. During the outbreak of the COVID-19 pandemic, most students rarely left their houses (81.76%), regularly washed their hands at least 20 s after sneezing/coughing and before eating (85.85%), avoided shaking hands, hugging or kissing others (78.05%), limited the use of public transport (83.51%), maintained a distance of at least one meter from others (73.95%), and avoided touching their eyes, noses, and mouths directly (75.32%). Almost all students (94.15%) usually wore face masks when having close contact/talking with others. However, 192 students conceded that there was a time they went out without wearing face masks (18.73%). In addition, although antibiotics do not work against the COVID-19 virus, 492 students (48.0%) still bought them for COVID-19 prevention. Four hundred ten students (40.0%) bought antiviral drugs (such as molnupiravir) to keep at home, although the VMOH announced that citizens should not store these medicines at home (Table 4). The average practice scores of 1,025 students were 9.18 ± 1.90 (range: 0–12).

**Table 4:** The practices of university students toward COVID-19 in the past month

No	Questions	“Yes” answers (%)
1	There was a time when I went out of the house without wearing a face mask	192 (18.73)
2	I usually avoided gathering/going out with friends and relatives	721 (70.34)
3	To limit the spread of COVID-19, I rarely left my house	838 (81.76)
4	I usually avoided touching my eyes, nose, and mouth directly	772 (75.32)
5	I regularly washed my hands with soap, alcohol, or hand sanitizer for at least 20 s after sneezing, coughing, and before eating	880 (85.85)
6	I usually wore a face mask when having close contact and talking with others	965 (94.15)
7	I usually avoided shaking hands, hugging, or kissing others	800 (78.05)
8	I usually maintained a distance of at least one meter from others	758 (73.95)
9	I limited my use of public transport	856 (83.51)
10	I bought antibiotics to prevent COVID-19	492 (48.00)
11	I bought antipyretics (such as paracetamol) and electrolyte balance solutions (such as oral rehydration salts) for backup	842 (82.15)
12	I bought antiviral drugs (molnupiravir, remdesivir) to keep at home	410 (40.00)

### The COVID-19 KAP scores of students and associated factors

A majority of students had good knowledge (75.61%), positive attitudes (98.24%), and good practices (94.93%). The average KAP scores of females and medical students were significantly higher than those of males and non-medical students, respectively (*p* < 0.001, *Wilcoxon rank-sum test*). The knowledge scores were positively correlated with the student's age and year of study (*p* < 0.001). There were no differences in the average KAP scores of students among provinces (*p* > 0.05, *Kruskal–Wallis test*). The average KAP scores of students living with their families (18.96 ± 4.35, 44.58 ± 4.01, 9.51 ± 1.88) were significantly higher than those of students living with their friends (17.53 ± 4.37, 43.89 ± 4.09, 8.93 ± 1.84) (*p* < 0.001, *p* = 0.0101, and *p* < 0.001), respectively (*Dunn test*). Furthermore, being infected with COVID-19 and participating in volunteer activities involving COVID-19 were not factors associated with the KAP scores of students (*p* > 0.05) (Table 5, Additional file 2).

**Table 5:** Factors associated with the COVID-19 knowledge, attitude, and practice scores of university students

No	Variables	Knowledge		Attitude		Practice	
		**Univariate**	**Multivariate**	**Univariate**	**Multivariate**	**Univariate**	**Multivariate**
		**Coef**	**Adj. Coef**	**Coef**	**Adj. Coef**	**Coef**	**Adj. Coef**
1	**Age** (continuous variable)	0.338***	0.204***	0.146***		0.110***	0.066***
2	**Sex** (ref.: Female)						
	Male	-1.658***		-2.003***	-1.515***	-0.595***	
3	**Year of study** (ref.: Fifth)						
	First	-3.415***		-1.034		-1.042***	
	Second	-2.338***		-0.894		-0.641*	
	Third	-0.109		0.601		0.268	0.583**
	Fourth	-0.064		0.541		0.403	
4	**Major** (Ref: Medical)						
	Non-medical	-3.495***	-2.808***	-1.775***	-1.337***	-1.045***	-0.766***
5	**Place of residence** (Ref: Rural)						
	Urban	0.971***		0.305		0.290*	
6	**Province** (Ref: Bacgiang)						
	Bacninh	-0.676		-0.531		-0.619	
	Haiduong	0.661		0.529		-0.605	
	Haiphong	-0.100		-0.122		-0.398	
	Hanoi	-0.035		0.005		-0.306	
	Hungyen	0.477		-0.156		-0.276	
	Namdinh	0.557		0.565		0.136	
	Thaibinh	0.135		1.000		-0.305	
	Thanhhoa	-0.126		-0.454		-0.517	
	Others	0.862		0.590		-0.300	
7	**The people living with the student** (Ref: Alone)						
	Family	1.312*		0.872		0.537*	
	Friends	-0.124		0.184		-0.044	
8	**Number of doses of COVID-19 vaccines given** (Ref: > 3)						
	No/one	-1.671		1.959		0.347	
	Two	-0.447		0.283		0.684	
	Three	-0.259		0.212		0.895	
9	**Infected with COVID-19** (Ref: No/Don’t know)						
	Yes	-0.018		-0.064		-0.167	
10	**Participated in COVID-19 volunteer activities in health facilities** (Ref: Already)						
	Never	-0.235		0.191		-0.191	
11	**Resources for seeking COVID-19 information**						
	**Social network/online newspapers** (Ref: No)						
	Yes	0.357		0.072		-0.267	
	**Family members/friends** (Ref: No)						
	Yes	0.680*		0.640*		0.088	
	**Mass media** (Ref: No)						
	Yes	1.128***		1.156***		0.140	
	**Websites of the World Health Organization, the Ministry of Health/scientific articles** (Ref: No)						
	Yes	1.349***	0.995***	1.393***	1.095***	0.485***	0.364**

Variables of the multivariate linear regression models were chosen by using the Bayesian Model Averaging method

Adjusted R-squared for the multivariate linear regression models: 0.1864 (Knowledge), 0.0938 (Attitude), and 0.109 (Practice)

*coef*. Coefficient, *adj. coef*. Adjusted coefficient, *ref*. Reference

^*** ^*p*-value < 0.001

^** ^*p*-value < 0.01

^*^ *p*-value < 0.05

Results from the multivariate linear regression models revealed that the student's major and using websites of WHO/VMOH and scientific articles for seeking COVID-19 information were two factors significantly associated with the KAP scores of students (*p* < 0.0014). Besides the two factors above, students' age was one factor significantly associated with their knowledge and practice scores (*p* < 0.001), while students' sex was the factor significantly associated with their attitude scores (*p* < 0.001). Older students were more likely to have higher COVID-19 knowledge and practice scores than the younger ones (*p* < 0.001). Furthermore, the study year was another factor significantly associated with students' practice scores. Third-year students were strongly associated with higher practice scores when compared with other students (*p* = 0.0056) (Table 5, Additional file 2).

## Discussion

This study was conducted during a wave of COVID-19 outbreaks in Vietnam. Schools and universities have been closed for a long time because of the detrimental impacts of this pandemic. The government strived to distribute COVID-19 vaccines nationwide and almost all people aged 18 years and above received at least two doses of vaccines [7]. Since April 2022, students have gradually come back to their universities. In the context of offline learning, students need to have adequate knowledge, positive attitudes, and good practices toward COVID-19. Our results showed that the KAP scores of a majority of Vietnamese university students were high. Despite being well aware of the main ways of COVID-19 transmission and symptoms, many students did not have adequate knowledge of COVID-19 treatment and prevention (WHO's mythbusters [40]) such as antibiotics, vitamins, and mineral supplements cannot treat or prevent COVID-19. In Italy, 19.30% of students thought that antibiotics were the first-line treatment for COVID-19 [27], far lower than the result of our study (42.73%). Wrong knowledge can lead to wrong actions/practices. Misusing antibiotics can contribute to the global increase of antibiotic resistance at an alarming rate in recent years. Another important finding was that females and medical students were significantly associated with higher KAP scores. In addition, several factors significantly associated with either the COVID-19 knowledge, attitudes, and/or practices of students included age, sex, year of study, major, and sources of COVID-19 information, in line with findings from studies in China [12], Indonesia [13–15], Pakistan [16, 17], Ethiopia [18], Bhutan [19], Serbia [20], Bangladesh [21], Malaysia [22], and Iran [23].

Three-quarters (75.61%) of Vietnamese students had good COVID-19 knowledge, lower than results from studies in Bhutan (98%) [19], Ecuador (88%) [24], Hochiminh, Vietnam (86.6%) [10], and Ethiopia (81.8%) [25] but far higher than results from studies in Indonesia (29.8%) [13], and China (28.3%) [12]. Regarding attitudes, almost all Vietnamese students (98.24%) had positive attitudes towards COVID-19, consistent with the findings of studies in Hochiminh, Vietnam (92.8%) [10], Pakistan (92.5%) [26], Italy (90.6%) [27], and Bhutan (86.6%) [19], but far higher than the results of studies in Ethiopia (70.9%) [25], China (67.8%, 73.81%) [12, 28], and Indonesia (64.9%, 55.7%) [13, 14]. The KAP scores varied across different countries [44]. These comparisons are only approximate because of the differences in the time for conducting studies, location, the number and difficulty level of questions, and the cut-off points for analyzing scores. By way of illustration, the authors of a study conducted in Indonesia used Bloom's cut-off (≥ 80%) to dichotomize the KAP scores [13], while a 50% cut-off point was used in our study. This could be the rationale behind the lower percentages in the former study.

Among the knowledge questions, there were four remarkable questions. Firstly, only 34.34% of Vietnamese students knew that exposure to/eating wild animals could not lead to COVID-19 infection (question K5), lower than the findings of studies in Serbia (49.1%) [20], Egypt (47.2%) [29], and Bhutan (44.6%) [19] but higher than those in Indonesia (31.8%) [13] and India (13.9%) [30]. 74.2% of university students in Pakistan knew that COVID-19 could not be transmitted via mosquito bites (question K4) [31]. This figure in our study was only 47.22%. In addition, only 37.17% of Vietnamese students did not choose antibiotics for COVID-19 treatment (question K13), far lower than the results from a study in Italy (80.70%) [27]. Furthermore, more than 70% of Vietnamese students thought that vitamins and minerals could help prevent or cure COVID-19 (question K16). The percentages of Vietnamese medical students having correct answers for questions K4, K5, K13, and K16 were 56.68, 36.84, 52.83, and 18.42%; significantly higher than those of non-medical students (38.42, 32.02, 22.60, and 9.04%), respectively (*p* < 0.001, except for question K5: *p* = 0.1187). In general, the course of study of non-medical students does not include subjects involving health. This can be the reason for their lower knowledge scores compared to medical students. Both non-medical and medical students participated in our study, while in other studies [10, 20, 24, 29], only the latter were enrolled. This can explain the lower percentages of students having correct answers in our study.

Two years have passed since the first COVID-19 outbreak in China. Vietnam's pandemic has gradually been controlled, but most university students (87.80%) still thought that COVID-19 was an extremely dangerous disease. This figure was in line with the results of studies in Bhutan (86.2%) [19], Indonesia (92.6%) [13], and Afghanistan (91.1%) [32]. In Serbia [20], the percentage of students getting nervous/anxious when watching/reading COVID-19 news was low (19.9%), while this figure for Vietnamese students was high (69.56%). About 32.78% of students thought that the government should not let overseas people return home during the outbreak of the COVID-19 pandemic, higher than the result of a Chinese study [33]. These negative attitudes can explain the students' high compliance with the COVID-19 prevention guidelines of the VMOH.

Vietnamese students' common sources for seeking information on COVID-19 included social network/online newspaper (87.61%), mass media (television/radio) (65.85%), websites of WHO/VMOH, and scientific articles (56.98%), consistent with the results of many other studies conducted in Ecuador (scientific articles: 77.3%) [24], Moroc (social networks: 67.9%) [34], Jordan (social media: 83.4%) [35], Egypt (social media: 75.7%) [36], Ethiopia (television: 83.8%) [18], and Turkey (television: 43%) [37]. Not only Vietnamese students but also students in other countries (Indonesia [13], Bhutan [19], Pakistan [21, 26, 31], Moroc [34], Jordan [35], and Palestine [38]) had many practical measures which could help to prevent COVID-19, such as avoiding touching eyes/noses/mouths directly, maintaining a distance of at least one meter from others, and avoiding gathering/going out with friends. These good practices can guarantee students' safety when they study and learn in their universities. Besides many good practices, since many Vietnamese students thought antibiotics could be used to treat COVID-19, 48.0% of them bought antibiotics for storage at home. Furthermore, although Vietnam has a policy of providing free antiviral medicines to COVID-19 patients, 40.0% of students still bought this type of medicine to keep at home to prevent COVID-19. These poor practices were mainly from non-medical students (buying antibiotics: medical students 36.03%, non-medical students 59.13% (*p* < 0.001); buying antiviral medicines: medical students 29.55%, non-medical students 49.72% (*p* < 0.001)). These findings indicated that non-medical students were the group that needed to be improved their COVID-19 knowledge and practices.

### Limitations

This research has some limitations. Firstly, this is only a cross-sectional study; therefore, the causal inferences among variables cannot be determined. In addition, using a self-reported questionnaire for collecting data can result in reporting bias/recall bias, and some questions can be dishonestly answered because of social desirability. Last but not least, by virtue of using a non-probability convenience sampling technique and only collecting data in one university, the sample cannot be generalizable. It cannot be extrapolated to the whole population of Vietnamese university students.

## Conclusions

Most Vietnamese university students had good knowledge, positive attitudes, and good practice toward COVID-19. However, many students did not know that antibiotics cannot be used to treat COVID-19 and vitamins/minerals cannot help prevent or cure COVID-19. Students' age, sex, year of study, major, and sources of COVID-19 information were several factors significantly associated with either the COVID-19 knowledge, attitudes, and/or practices of students. The leaders of Phenikaa University should focus on student groups having low KAP scores, such as non-medical students and males, to enhance their KAP toward COVID-19, thereby guaranteeing the students' safety in the context of offline learning.

## Supplementary Information

**Additional file 1.** The questionnaire. 

**Additional file 2.** The mean knowledge, attitude, and practice scores of university students regarding COVID-19.

## Data Availability

The datasets used and/or analyzed during the current study are available from the corresponding author upon reasonable request.

## Abbreviations

COVID-19: Coronavirus disease 2019
WHO: World Health Organization
VMOH: Vietnam Ministry of Health
KAP: Knowledge, attitudes, practices
