# Supplementary material for: Knowledge, attitudes, and practices of university students regarding COVID-19: a cross-sectional study in Vietnam
Source: BMC Public Health. 2022 Nov 3;22:2016. doi: 10.1186/s12889-022-14442-9 (PMC9633125; doi:10.1186/s12889-022-14442-9)
Supplement: Supplementary file 1 — Additional file 1. The questionnaire. [file 12889_2022_14442_MOESM1_ESM.pdf]

Additional file 1. SURVEYING THE KNOWLEDGE, ATTITUDE, AND PRACTICES OF UNIVERSITY STUDENTS REGARDING COVID-19

Dear students. The research team from Phenikaa University is conducting a study to survey university students' knowledge, attitude, and practices regarding the COVID-19 pandemic in Vietnam. The time for answering questions is about 10 to 15 minutes. All information in this data collection form is only used for studying. We hope that you will answer questions fully and honestly. The identity of participants will be kept confidential. We truly appreciate your participation and contribution to our research.

Do you agree to take part in this survey?

☐ Yes ☐ No

No	Questions	Answers
**Students' information**		
I1	Sex	☐ Male<br>☐ Female
I2	Age	
I3	Province (home location)	Province: ....
I4	Place of residence	☐ Urban<br>☐ Rural
I5	Year of study	☐ First<br>☐ Second<br>☐ Third<br>☐ Fourth<br>☐ Fifth
I6	Name of faculty/department	....
I7	The people who are living with you	☐ My family (grandparents, parents, husband/wife, siblings...)<br>☐ Relatives (aunts, uncles...)<br>☐ Friends<br>☐ Alone<br>☐ Others: ....<br>...<br>...
I8	Have you ever been infected with COVID-19?	☐ Yes<br>☐ No<br>☐ I don't know
I9	Have you ever participated in COVID-19 volunteer activities in health facilities?	☐ Already<br>☐ Never
I10	The number of doses of COVID-19 vaccines given	....

No	Questions	Answers
I11	Your sources for seeking COVID-19 information	☐ Social networks (such as Facebook, Messenger, Zalo apps...), and online newspapers<br>☐ Friends, family members<br>☐ Mass media (national news, radio, television...)<br>☐ Websites of the World Health Organization and the Ministry of Health, and scientific articles<br>☐ Others: ....<br>..<br>..<br>..<br>..
**B. Knowledge**		
K1	COVID-19 is a disease caused by the SARS-Cov-2 virus.	☐ Totally right<br>☐ Probably right<br>☐ Don’t know<br>☐ Probably wrong<br>☐ Totally wrong
K2	The virus can be transmitted through droplets produced when an infected person coughs, sneezes, or talks.	☐ Totally right<br>☐ Probably right<br>☐ Don’t know<br>☐ Probably wrong<br>☐ Totally wrong
K3	You can be infected with COVID-19 when touching contaminated surfaces, not washing your hands, and then touching your eyes, nose, or mouth.	☐ Totally right<br>☐ Probably right<br>☐ Don’t know<br>☐ Probably wrong<br>☐ Totally wrong
K4	The COVID-19 virus can be spread through mosquito bites.	☐ Totally right<br>☐ Probably right<br>☐ Don’t know<br>☐ Probably wrong<br>☐ Totally wrong
K5	Exposure to or eating wild animals can lead to COVID-19 infection.	☐ Totally right<br>☐ Probably right<br>☐ Don’t know<br>☐ Probably wrong<br>☐ Totally wrong

No	Questions	Answers
K6	PCR (Polymerase Chain Reaction) can be used to accurately diagnose whether or not a person is infected with COVID-19.	☐ Totally right<br>☐ Probably right<br>☐ Don’t know<br>☐ Probably wrong<br>☐ Totally wrong
K7	Symptoms of COVID-19 infection can include fever, cough, fatigue, shortness of breath, and loss of taste/smell.	☐ Totally right<br>☐ Probably right<br>☐ Don’t know<br>☐ Probably wrong<br>☐ Totally wrong
K8	Unlike the common cold, runny nose and sneezing are less common in COVID-19 patients.	☐ Totally right<br>☐ Probably right<br>☐ Don’t know<br>☐ Probably wrong<br>☐ Totally wrong
K9	A person can be infected with COVID-19 without any unusual symptoms.	☐ Totally right<br>☐ Probably right<br>☐ Don’t know<br>☐ Probably wrong<br>☐ Totally wrong
K10	Patients with chronic medical conditions, the elderly, and the obese have a lower risk of mortality and milder symptoms of COVID-19 infection.	☐ Totally right<br>☐ Probably right<br>☐ Don’t know<br>☐ Probably wrong<br>☐ Totally wrong
K11	Washing hands with soap, using face masks, and limiting crowded gatherings are some effective measures to help prevent COVID-19.	☐ Totally right<br>☐ Probably right<br>☐ Don’t know<br>☐ Probably wrong<br>☐ Totally wrong
K12	Hydroxychloroquine may help prevent infection or death from COVID-19.	☐ Totally right<br>☐ Probably right<br>☐ Don’t know<br>☐ Probably wrong<br>☐ Totally wrong
K13	Antibiotics are the first choice to treat COVID-19.	☐ Totally right<br>☐ Probably right<br>☐ Don’t know<br>☐ Probably wrong<br>☐ Totally wrong

No	Questions	Answers
K14	Adding pepper, ginger, and garlic to food does not help prevent and treat COVID-19.	☐ Totally right<br>☐ Probably right<br>☐ Don't know<br>☐ Probably wrong<br>☐ Totally wrong
K15	Drinking alcohol and exposure to sunlight/temperatures higher than 25°C can help prevent COVID-19.	☐ Totally right<br>☐ Probably right<br>☐ Don't know<br>☐ Probably wrong<br>☐ Totally wrong
K16	Vitamins and minerals can help prevent COVID-19 in healthy people or cure COVID-19 in infected people.	☐ Totally right<br>☐ Probably right<br>☐ Don't know<br>☐ Probably wrong<br>☐ Totally wrong
**C. Attitude**		
A1	COVID-19 is an extremely dangerous disease.	☐ Totally agree<br>☐ Agree<br>☐ Neutral<br>☐ Disagree<br>☐ Totally disagree
A2	I feel very anxious when watching or reading the news about COVID-19.	☐ Totally agree<br>☐ Agree<br>☐ Neutral<br>☐ Disagree<br>☐ Totally disagree
A3	I feel apprehensive and insecure if I, my friends, or my family members are infected with COVID-19.	☐ Totally agree<br>☐ Agree<br>☐ Neutral<br>☐ Disagree<br>☐ Totally disagree
A4	Children and adolescents do not need to take any measures to prevent COVID-19.	☐ Totally agree<br>☐ Agree<br>☐ Neutral<br>☐ Disagree<br>☐ Totally disagree
A5	I will always comply with the COVID-19 prevention guidelines of the Ministry of Health.	☐ Totally agree<br>☐ Agree<br>☐ Neutral<br>☐ Disagree

No	Questions	Answers
		☐ Totally disagree
A6	Wearing face masks is very effective in preventing COVID-19.	☐ Totally agree<br>☐ Agree<br>☐ Neutral<br>☐ Disagree<br>☐ Totally disagree
A7	If infected with the virus, I am ready to go to quarantine at medical facilities or self-isolate at home in case my symptoms are mild.	☐ Totally agree<br>☐ Agree<br>☐ Neutral<br>☐ Disagree<br>☐ Totally disagree
A8	When a person is infected with COVID-19, making a medical declaration is really necessary.	☐ Totally agree<br>☐ Agree<br>☐ Neutral<br>☐ Disagree<br>☐ Totally disagree
A9	I am willing to volunteer at medical facilities to support the fight against the COVID-19 pandemic if necessary.	☐ Totally agree<br>☐ Agree<br>☐ Neutral<br>☐ Disagree<br>☐ Totally disagree
A10	The government should not let overseas Vietnamese return home during the outbreak of the COVID-19 pandemic.	☐ Totally agree<br>☐ Agree<br>☐ Neutral<br>☐ Disagree<br>☐ Totally disagree
A11	Updating COVID-19 information regularly to know how to prevent this disease and avoid misinformation is really necessary and important.	☐ Totally agree<br>☐ Agree<br>☐ Neutral<br>☐ Disagree<br>☐ Totally disagree
A12	I think the COVID-19 pandemic will be successfully controlled soon.	☐ Totally agree<br>☐ Agree<br>☐ Neutral<br>☐ Disagree<br>☐ Totally disagree
**D. Practices**<br>In the past month...		
P1	There was a time when I went out of the house without wearing a face mask.	☐ Yes<br>☐ No

No	Questions	Answers
P2	I usually avoided gathering/going out with friends and relatives.	☐ Yes<br>☐ No
P3	To limit the spread of COVID-19, I rarely left my house.	☐ Yes<br>☐ No
P4	I usually avoided touching my eyes, nose, and mouth directly.	☐ Yes<br>☐ No
P5	I regularly washed my hands with soap, alcohol, or hand sanitizer for at least 20 seconds after sneezing, coughing, and before eating.	☐ Yes<br>☐ No
P6	I usually wore a face mask when I had close contact and talked with others.	☐ Yes<br>☐ No
P7	I usually avoided shaking hands, hugging, or kissing others.	☐ Yes<br>☐ No
P8	I usually maintained a distance of at least one meter from others.	☐ Yes<br>☐ No
P9	I limited my use of public transport.	☐ Yes<br>☐ No
P10	I bought antibiotics to prevent COVID-19.	☐ Yes<br>☐ No
P11	I bought antipyretics (such as paracetamol) and electrolyte balance solutions (such as oral rehydration salts) for backup.	☐ Yes<br>☐ No
P12	I bought antiviral drugs (such as molnupiravir) to keep at home.	☐ Yes<br>☐ No

Thank you for assisting us in data collection.

Best regards.
