# Supplementary material for: Knowledge, attitudes, and practices of university students regarding COVID-19: a cross-sectional study in Vietnam
Source: BMC Public Health. 2022 Nov 3;22:2016. doi: 10.1186/s12889-022-14442-9 (PMC9633125; doi:10.1186/s12889-022-14442-9)
Supplement: Supplementary file 2 — Additional file 2. The mean knowledge, attitude, and practice scores of university students regarding COVID-19. [file 12889_2022_14442_MOESM2_ESM.pdf]

Additional file 2.

The mean knowledge, attitude, and practice scores of university students regarding COVID-19

No.	Characteristics		Number	Knowledge		Attitude		Practice	
				Mean (SD)	p-value	Mean (SD)	p-value	Mean (SD)	p-value
1	Sex	Male	312	17.01 (4.55)	<0.0001	42.79 (4.39)	<0.0001	8.77 (1.97)	<0.0001
		Female	713	18.67 (4.44)		44.79 (3.79)		9.36 (1.84)	
2	Age	18	245	17.06 (4.59)	<0.0001	43.93 (3.98)	0.00187	8.67 (1.86)	<0.0001
		19	366	17.31 (4.00)		43.91 (3.91)		8.94 (1.91)	
		20	189	18.46 (4.66)		43.79 (4.03)		9.35 (1.75)	
		21	70	19.47 (4.59)		44.66 (4.15)		9.76 (1.83)	
		22 and above	155	20.97 (4.12)		45.46 (4.47)		10.11 (1.73)	
3	Year of study	First	570	17.21 (4.33)	<0.0001	43.85 (4.00)	0.00145	8.82 (1.90)	<0.0001
		Second	242	18.29 (4.45)		43.99 (3.88)		9.22 (1.80)	
		Third	83	20.52 (3.92)		45.48 (4.15)		10.13 (1.57)	
		Fourth	71	20.56 (4.77)		45.42 (4.59)		10.27 (1.67)	
		Fifth	59	20.63 (4.25)		44.88 (4.39)		9.86 (1.87)	
4	Major	Medical	494	19.97 (3.99)	<0.0001	45.10 (3.94)	<0.0001	9.72 (1.78)	<0.0001
		Non-medical	531	16.48 (4.37)		43.33 (4.03)		8.68 (1.87)	
5	Place of residence	Urban	408	18.75 (4.64)	<0.0001	44.37 (4.27)	0.1847	9.36 (1.86)	0.01554
		Rural	617	17.78 (4.43)		44.06 (3.96)		9.07 (1.92)	

No.	Characteristics		Number	Knowledge		Attitude		Practice	
				Mean (SD)	p-value	Mean (SD)	p-value	Mean (SD)	p-value
6	Province (home location)	Hanoi	305	17.83 (4.96)	0.2521	43.95 (4.17)	0.2422	9.18 (1.87)	0.4315
		Namdinh	90	18.42 (4.51)		44.51 (4.02)		9.62 (1.64)	
		Thanhhoa	65	17.74 (4.93)		43.49 (3.73)		8.97 (1.57)	
		Haiduong	59	18.53 (4.09)		44.47 (4.26)		8.88 (2.27)	
		Thaibinh	55	18.00 (4.23)		44.95 (4.09)		9.18 (2.06)	
		Bacninh	53	17.19 (4.50)		43.42 (4.18)		8.87 (2.17)	
		Hungyen	38	18.34 (3.69)		43.79 (3.65)		9.21 (1.79)	
		Bacgiang	37	17.86 (3.71)		43.95 (3.99)		9.49 (1.54)	
		Haiphong	34	17.76 (4.54)		43.82 (4.55)		9.09 (1.88)	
		Others	289	18.73 (4.31)		44.54 (4.03)		9.19 (1.95)	
7	The people living with the student	Alone	69	17.65 (5.97)	<0.0001	43.71 (4.36)	0.00881	8.97 (2.10)	<0.0001
		Friends	509	17.53 (4.37)		43.89 (4.09)		8.93 (1.84)	
		Family (such as parents, siblings...)	447	18.96 (4.35)		44.58 (4.01)		9.51 (1.88)	
8	Number of doses of COVID-19 vaccines given	No or 1	10	16.80 (5.71)	0.433	45.90 (4.15)	0.6368	8.70 (2.79)	0.1178
		2	214	18.02 (4.45)		44.22 (4.30)		9.04 (1.87)	
		3	784	18.21 (4.55)		44.15 (4.00)		9.25 (1.88)	
		>3	17	18.47 (4.67)		43.94 (5.38)		8.35 (2.34)	

No.	Characteristics			Number	Knowledge		Attitude		Practice	
					Mean (SD)	p-value	Mean (SD)	p-value	Mean (SD)	p-value
9	Infected with COVID-19	Yes		616	18.16 (4.48)	0.9818	44.16 (4.11)	0.5475	9.12 (1.89)	0.1239
		No/don't know		409	18.17 (4.62)		44.22 (4.05)		9.28 (1.91)	
10	Participated in COVID-19 volunteer activities in health facilities	Already		192	18.35 (4.61)	0.3788	44.03 (4.11)	0.4308	9.34 (1.82)	0.2964
		Never		833	18.12 (4.52)		44.22 (4.08)		9.15 (1.91)	
11	Sources for seeking COVID-19 information	Social networks (Facebook, Zalo apps...), online newspapers	Yes	898	18.21 (4.47)	0.6265	44.19 (4.03)	0.9286	9.15 (1.90)	0.1269
			No	127	17.85 (5.00)		44.12 (4.46)		9.42 (1.83)	
		Friends, family members	Yes	559	18.47 (4.32)	0.03404	44.47 (3.85)	0.01078	9.22 (1.85)	0.5345
			No	466	17.79 (4.76)		43.83 (4.33)		9.14 (1.96)	
		Mass media (national news, radio, television)	Yes	675	18.55 (4.37)	0.00031	44.58 (3.87)	<0.0001	9.23 (1.90)	0.2418
			No	350	17.42 (4.76)		43.42 (4.38)		9.09 (1.89)	
		Websites of the World Health Organization, the Ministry of Health, and scientific articles	Yes	584	18.74 (4.42)	<0.0001	44.78 (3.85)	<0.0001	9.39 (1.89)	<0.0001
			No	441	17.39 (4.58)		43.39 (4.25)		8.91 (1.87)	
